# Supplementary material for: Interpretable, non-mechanistic forecasting using empirical dynamic modeling and interactive visualization
Source: PLoS One. 2023 Apr 3;18(4):e0277149. doi: 10.1371/journal.pone.0277149 (PMC10069763; doi:10.1371/journal.pone.0277149)
Supplement: S1 File — Comparison of the elements of the method with some basic benchmarks. (PDF) [file pone.0277149.s001.pdf]

Supporting Information S1

The standard way to compare the accuracy of forecasting methods is by calculating some error metric (such as root mean-square error or mean absolute error) between the point forecasts and the true observed values. It is difficult to assess EpiForecast in this way because the tool is designed to augment point forecasts with more abstract ideas such as interpretability, interaction, and detailed visualization. However, it is useful to get a grasp on how certain elements of the tool compare to basic benchmarks. To this end, we have provided a notebook which shows how the distributional accuracy of the kernel density estimation method compares to a typical normal distribution-based approach. The notebook also shows how the peaks of the estimated distribution compare to the actual values. This notebook is found at https://observablehq.com/@siliconjazz/epiforecast-dist-accuracy . In this document we provide a copy of the tool's results for EpiForecast's default dataset: mortality data from the CDC.

Horizon	Mean KDE Score	Mean Normal Score	Difference
1	9.215	9.404	-0.189
2	9.374	9.629	-0.255
3	9.530	9.782	-0.252
4	9.693	9.952	-0.259
5	9.939	10.101	-0.162
6	10.145	10.193	-0.047
7	10.223	10.278	-0.055
8	10.287	10.358	-0.071
9	10.362	10.462	-0.101
10	10.576	10.802	-0.226
11	10.628	10.827	-0.199
12	10.692	11.000	-0.308
13	10.733	11.103	-0.369
14	10.764	11.221	-0.457
15	10.760	11.210	-0.450
16	10.726	11.188	-0.461
17	10.830	11.193	-0.362
18	10.832	11.239	-0.407
19	10.900	11.305	-0.405
20	10.963	11.376	-0.413
21	10.773	11.127	-0.354
22	10.646	10.822	-0.176
23	10.612	10.634	-0.022

24	10.641	10.520	0.121
25	10.567	10.399	0.168
26	10.452	10.295	0.156
27	10.360	10.183	0.177
28	10.250	10.104	0.146
29	10.119	10.038	0.081
30	10.026	9.890	0.137
31	9.961	9.757	0.204
32	9.958	9.785	0.173

Table 1: Tabular output of the supplementary accuracy comparison notebook, on the default data (mortality from the CDC). The 'score' here is the logarithmic score, a popular metric for comparing distributional forecasts (1). The distributional KDE score is compared with a benchmark, normal-distribution method.

Horizon	Mean Simplex MAE	Mean Best Peak MAE	Mean Worst Peak MAE
1	1289.2	1201.3	2113.4
2	1389.0	1286.3	2406.9
3	1524.9	1310.5	2683.1
4	1672.4	1360.8	2886.3
5	1791.4	1432.9	3105.4
6	1909.3	1469.2	3331.9
7	2021.6	1505.3	3515.1
8	2130.9	1645.0	3652.8
9	2200.5	1612.0	3786.3
10	2260.4	1824.0	3875.5
11	2278.1	1798.9	3851.1
12	2349.8	1769.5	3842.8
13	2400.6	1812.1	3881.4
14	2459.2	1835.4	3884.3
15	2517.0	1862.0	3914.6
16	2599.7	1865.9	4007.1
17	2651.6	1862.1	4079.8
18	2704.6	1864.5	4132.0
19	2731.5	1853.5	4130.4
20	2727.3	1917.0	4068.5
21	2682.5	1854.2	4076.2
22	2650.3	1846.0	4080.1
23	2576.4	1812.3	4084.3
24	2467.6	1848.0	4077.0
25	2354.6	1689.8	3981.0
26	2260.6	1660.4	3904.8
27	2161.4	1601.7	3733.2
28	2103.7	1611.5	3722.3
29	2054.5	1556.4	3575.8
30	2025.4	1524.3	3485.9
31	1972.9	1497.7	3418.3
32	1957.4	1415.7	3594.3

Table 2: Tabular output of the supplementary accuracy comparison notebook, on the default data (mortality from the CDC). The mean absolute error (MAE) of the best and worst peaks from the kernel density estimation (KDE) method are compared with the point forecasts generated using the simplex method.

References

1. Jordan A, Krüger F, Lerch S. Evaluating probabilistic forecasts with scoringRules [Internet]. arXiv; 2018 [cited 2023 Feb 23]. Available from: http://arxiv.org/abs/1709.04743
