# Supplementary material for: Interpretable, non-mechanistic forecasting using empirical dynamic modeling and interactive visualization
Source: PLoS One. 2023 Apr 3;18(4):e0277149. doi: 10.1371/journal.pone.0277149 (PMC10069763; doi:10.1371/journal.pone.0277149)
Supplement: Attachment — Submitted filename: revew2.pdf [file pone.0277149.s002.pdf]

Thursday 19 January 2023

Review of “Interpretable, non-mechanistic forecasting using empirical dynamic modeling and interactive visualization”

Manuscript Number: PONE-D-22-28854

The authors provided a web-application as a tool to produce the interpretations of non-mechanistic forecasts using Empirical Dynamic Modelling and interactive visualization. This tool is based on empirical dynamic modelling (EDM), a simple and intuitive non-mechanistic technique with four interactive plots. They made this tool available online for researchers and more details. Results, discussions and the statistical method used have been introduced. The manuscript is technical without theoretical contribution. Novelty resides in providing a web-application.

In my opinion, the manuscript is interesting idea and scientifically coherent. It tackles an important issue. The state of the literature in the background is comprehensive and the related work is adequately cited. However, I find :

- Difficulty in following all the information in the manuscript due to the presentation is not very clear to me.
- The proposed tool should represent by an algorithm. It is not easy to understand the methodology of the tool through textual narration.
- I encourage the authors to spend more time in the section on Methods to present the details through a mathematical framework, as well as, for the mathematical notations.
- The authors full name in the reference should be included. For example, in reference (11) there are missing authors' names.
- Finally, the details of interactive visualization are not presented as well.

To conclude, It is an interesting work that would deserve to be published in your journal.
