# Supplementary material for: Interpretable, non-mechanistic forecasting using empirical dynamic modeling and interactive visualization
Source: PLoS One. 2023 Apr 3;18(4):e0277149. doi: 10.1371/journal.pone.0277149 (PMC10069763; doi:10.1371/journal.pone.0277149)
Supplement: Attachment — Submitted filename: Response To Reviewers.docx [file pone.0277149.s003.docx]

Dear Dr. Algamal,

Thank you for inviting us to re-submit our work to PLOS ONE. We would also like to thank you and the reviewers for taking the time to consider our manuscript and provide helpful feedback. We are very grateful for the insightful comments raised by the reviewers, and we hope that the revised manuscript is suitable for publication in your journal. Below, we have listed the suggestions and the changes we made in response:

- *“Difficulty in following all the information in the manuscript due to the presentation is not very clear to me.” (Reviewer 2)*

We have updated several parts of the manuscript to improve clarity, especially the methods and results sections.

- *“The proposed tool should represent by an algorithm. It is not easy to understand the methodology of the tool through textual narration.” (Reviewer 2)*
- *“I encourage the authors to spend more time in the section on Methods to present the details through a mathematical framework, as well as, for the mathematical notations.” (Reviewer 2)*
- *“Methods section doesn't have sufficient details and basic equations about proposed methods.” (Reviewer 1)*

In the methods section, we have moved many of the inline equations to their own lines, and updated the surrounding narration to clarify the role each equation plays in the tool.

- *“Finally, the details of interactive visualization are not presented as well.” (Reviewer 2)*

A very helpful comment, and we agree completely that a specific account of the interactive plots was missing in the original manuscript. To remedy this, we have re-written and expanded much of the results section, specifically to include more details of the interaction.

- *“The authors full name in the reference should be included. For example, in reference (11) there are missing authors' names.” (Reviewer 2)*

The references in our report are in the Vancouver style, as suggested by the PLOS ONE guidelines. The Vancouver style has a unique way of representing authors name where their last name is followed by their initials, without periods between the initials (e.g. for me it would be Mason LK). We (myself and another co-author) were unfamiliar with this style prior to submission but we have re-checked the references and we believe they adhere to this style. Regarding reference (11) in particular, the name “Pe’er G” perhaps appears abbreviated, but that is the spelling listed on the paper.

- *“Abstract should be rewritten correctly with more details about proposed and used methods.” (Reviewer 1)*

We have re-written the abstract to include a specific reference to the methods used (empirical dynamic modelling) and the methods proposed (the interactive dashboard and the shaded forecasting area based on kernel density estimation).

- *“In results section, statistical error measurements should be used for comparing the accuracy of result and inserted into tables.” (Reviewer 1)*

In the results section, we have now included a link to a notebook which compares the accuracy of the kernel density estimation method to some basic benchmarks. We have also included a tabular form of these comparisons (for the default data) in Supporting Information S1; this is also referenced in the results section.

- *Conclusions should be rewritten in more appropriate contents. (Reviewer 1)*

We have rewritten the conclusion paragraph so that it relates better to the rest of the manuscript.

Once again, we’d like to thank the reviewers for these helpful comments. We are delighted to have to resubmit this paper with the suggested changes. We look forward to hearing your response.

Sincerely,

Lee Mason

Division of Cancer Epidemiology and Genetics, National Cancer Institute 9609 Medical Center Drive, Rockville, Maryland 20850

Email: masonlk@nih.gov
